# Electronic Structure of Rh and Ir Single Atom Catalysts Supported on Defective and Doped ZnO: Assessment of Their Activity Towards CO Oxidation

**DOI:** 10.3390/molecules29215082

**Published:** 2024-10-28

**Authors:** Arda Erbasan, Hande Ustunel, Daniele Toffoli

**Affiliations:** 1 Department of Physics, Middle East Technical University, Dumlupinar Blv 1, Ankara 06800, Turkey; arda.erbasan@metu.edu.tr; 2 Dipartimento di Scienze Chimiche e Farmaceutiche, Università degli Studi di Trieste, Via L. Giorgieri 1, 34127 Trieste, Italy; 3 IOM-CNR, Istituto Officina dei Materiali-CNR, S.S.14, Km 163.5, 34149 Trieste, Italy

**Keywords:** density functional theory, single atom catalysis, CO activation, O_2_ activation, oxidation reaction

## Abstract

This study investigated the electronic structure of single-atom Rhodium (Rh) and Iridium (Ir) adsorbed on defective and impurity-doped ZnO(0001) surfaces, and assessed their activity towards the CO oxidation reaction. Our findings reveal that surface impurities significantly influence the binding energies and electronic properties of the metal atoms, with Al and Cr serving as particularly effective promoters. While Rh and Ir acquire a positive charge upon incorporation on the unpromoted Zn(0001) surface, adsorption directly on the promoter results in a net negative charge, thus facilitating the activation of both CO and O_2_ species. These results highlight the potential of impurity-promoted ZnO surfaces in modulating and tailoring the electronic properties of SACs, which can be used for a rational design of active single-atom catalysts.

## 1. Introduction

Single-atom catalysts (SACs) represent the upper limit of surface-to-volume ratio achievable by the metal component of a heterogeneous catalyst while offering higher resistance against erosion and poisoning than their larger counterparts [1]. SACs, on the other hand, are susceptible to facile diffusion and loss of reactivity via segregation [2]. In general [3,4,5], a complex relation between the metal atoms and the support determines the stability, activity, and selectivity of the metal atom for a given reaction [6,7,8,9].

Transition metal atoms dispersed on oxide supports are a prevalent category of SACs [10]. Oxide supports are frequently employed due to their cost-effectiveness, stability, and the abundance of active sites on their surfaces. In addition, oxide supports feature various defects, such as oxygen and metal vacancies, edges, steps, and terraces, which serve as anchor sites for single metals [11,12]. This characteristic has been utilized in various catalyst designs. For instance, Zhou et al. [13] showed that the surface arrangement of Pt single atoms could be controlled by modifying the coordination environment on TiO_2_ supports using defect engineering techniques. Similarly, stabilizing single-atom sites via oxygen vacancies was shown to increase the performance of a Rh-SnO_2_ catalyst towards the gas-phase hydroformylation of ethylene [14]. Beyond oxygen vacancies, cation vacancies also function as effective anchor sites. As an example, stabilizing single metals by lattice oxygens in a metal vacancy site has been demonstrated to increase the efficacy of Pt-CrO_2_ [15] and Pt/Au-ZnO [16] towards wet CO oxidation and methanol steam reforming, respectively. Furthermore, metal oxides can readily exchange electrons with metals, potentially improving their reactivity and selectivity by causing a shift of their *d* orbitals [17]. Shi et al. [18] reported that *d* orbital occupation of a Au atom placed on oxygen-deficient TiO_2_ can be controlled through substrate doping, thereby enhancing its catalytic efficiency.

In addition to employing vacancy-type defects, the performance of SACs can be further tuned by introducing a second metal as a promoter. For instance, Searles et al. demonstrated that silica-supported Ga-Pt nanoparticles generated from single-atom sites are highly effective and stable towards propane dehydrogenation [19]. Other notable examples include silica-supported Pt-Zn [20] and silica-supported Pt-Ti [21], which are also used for propane dehydrogenation.

This study investigates single Rh and Ir atoms supported on defective and metal-decorated ZnO(0001) surfaces for CO oxidation. Specifically, it examines how surface impurities, acting as promoters, influence their electronic properties and, as a consequence, their catalytic properties. ZnO has attracted significant attention in the last few years as a support for SACs thanks to its stability, optical properties, and nontoxicity [22,23,24,25]. In the context of SACs, the ability to stabilize metal atoms through the creation of surface defects has been shown to yield highly efficient catalysts. For instance, a theoretical study by Jacobo-Fernandez et al. proposed magnetic SACs with Pt and Pd on defective ZnO surfaces as an efficient catalyst for the oxygen reduction reaction [25]. Mohite et al. designed and measured the performance as photocatalysts of single Au atoms on defective ZnO surfaces [26]. In a different geometric configuration, ZnO quantum dots with transition metal single atoms (Fe, Co, Ni, Cu) have been used as catalysts for the conversion of glycerol to glycerol carbonate [23].

Our choice of Rh and Ir as the metal atoms is largely motivated by the fact that there have not been many studies combining their well-known catalytic properties and the advantages of ZnO as a support. Rh, in particular, has been extensively investigated with other supports including Al_2_O_3_ [27,28], CeO_2_ [29,30], phosphotungstic acid [31,32,33], graphdiyne [34], TiO_2_ [35,36], ${\mathrm{FeO}}_{x}$ [37], and ZnO nanowires (nw) [22]. Notably, Han et al. [22] demonstrated that Rh supported on ZnO-nw exhibits very high catalytic activity toward dry CO oxidation reaction compared to Au and Pt single metals. Rh has also been investigated for reactions such as hydroformylation [38,39,40,41], formic acid electro-oxidation [42], and CO_2_ reduction [43]. Lang et al. further confirmed that Rh SAC supported by ZnO-nw shows excellent catalytic activity and stability for the hydroformylation of olefins [41].

In contrast, there are fewer studies on Ir as a single metal [44,45,46,47,48,49]. However, there are some examples where Rh and Ir were investigated together for CO oxidation on hexagonal boron nitride nanosheets [50], zeolites [51], GeS monolayer [52], MgO and Al_2_O_3_ [53,54], and polyoxometalate [55]. A few examples in which Rh and Ir were dispersed on a ZnO support can be found [7,56,57].

The CO oxidation reaction is widely recognized as a benchmark for evaluating catalytic activity [58,59]. To facilitate the investigation of reactivity, several descriptors have been employed, including the adsorption energies of reactants and products [60,61], partial density of states (PDOS) profiles and *d*-band centers [62], partial charges and charge transfer [63], and molecular bond lengths and stretching mode frequencies [64]. In this study, we applied these descriptors to assess the catalytic potential of Rh and Ir single atoms toward CO oxidation on defective and promoted ZnO(0001) surfaces. In selecting the promoters, a diverse range was considered: Al represented low-valent metals, Cr was a magnetic impurity, Pd was included as a noble metal, and Cu was chosen for its well-known reactivity.

The plan of the paper is as follows: In Section 2, we present the computational details. Section 3 collects our results along with their interpretation and analysis. Section 4 summarizes the outcomes of this work.

## 2. Computational Details

All calculations were performed within the density functional theory (DFT) framework as implemented in the Vienna Ab Initio Simulation Package (VASP) [65,66,67]. Electronic exchange and correlation was described using the Perdew–Burke–Ernzerhof (PBE) formalism [68] while electron–ion interactions were described by the projector-augmented-wave (PAW) scheme [69,70]. 3d-4s, 2s-2p, 2s-2p, 4d-5s, 5d-6s, 3s-3p, 3d-4s, 3d-4s, and 4d-5s electrons of Zn, O, C, Rh, Ir, Al, Cr, Cu, and Pd were included in the calculations as valence electrons, respectively. Geometry optimization was carried out until all forces reached 0.01 eV/Å with an electron energy convergence of $10^{-5}$ eV. All calculations were performed with spin polarization. The plane-wave basis was truncated using a kinetic energy cutoff of 600 eV. A Hubbard correction was employed with $U_{eff}=5$ eV for the on-site Coulomb interaction on Zn-*d* orbitals as described by Dudarev et al. [71]. This choice for the $U_{eff}$ was motivatived by its ability to give lattice constants in closer agreement with experimentally determined lattice constants compared to other theoretical studies using different $U_{eff}$ values for ZnO [72].

The Zn-terminated ZnO(0001) surface was modeled using six-layer slabs with 2×2 and 3×3 periodically repeating surface units to test for coverage effects. Γ-centered Monkhorst-Pack grids of 11×11×1 and 7×7×1 were employed for Brillouin zone integrations for the 2×2 and 3×3 cells, respectively. A minimum vacuum spacing of 14.5 Å was employed for all calculations to reduce interactions between adjacent periodic images. Geometric optimization of the surfaces resulted in the inward relaxation of top layers, as also noted in the literature [73].

Some descriptors used in this work to assess reactivity were adsorption energies, charge density differences upon adsorption, bond lengths, bond stretching mode frequencies, partial charges, and partial density of states (PDOS) profiles. Adsorption energies were calculated using

(1) $$E_{b}=E_{tot}-E_{surf}-E_{ads}$$

where $E_{tot}$, $E_{surf}$, and $E_{ads}$ are the electronic ground state energies of the system, the surface, and the isolated adsorbate, respectively. With this definition, negative energies indicate stable adsorption. Charge density difference (CDD) contours were calculated in a similar way via the expression

(2) $$\Delta \rho \left(\vec{r}\right)=\rho_{tot}\left(\vec{r}\right)-\rho_{surf}\left(\vec{r}\right)-\rho_{ads}\left(\vec{r}\right)$$

where $\rho_{tot}\left(\vec{r}\right)$, $\rho_{surf}\left(\vec{r}\right)$, and $\rho_{ads}\left(\vec{r}\right)$ are the electronic ground state density of the system, the surface, and the isolated adsorbate, respectively. Vibrational frequencies were calculated by diagonalizing the force constant matrix obtained by central finite differences with a step size of 0.01 Å. During the calculation of the frequencies, the adsorbing species as well as the metals were allowed to move. Finally, Bader decomposition was used to calculate partial charges using the software developed by the Henkelman (version 1.05) group [74].

## 3. Results and Discussion

### 3.1. Substitutional Incorporation of Rh and Ir Atoms on the ZnO(0001) Surface

**Figure 1:**
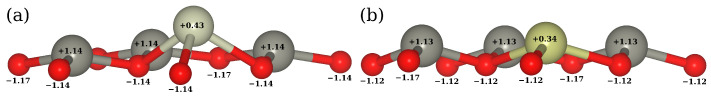
Optimized geometry and Bader charges on the Rh (**a**) and Ir (**b**) atoms in substitutional positions on the ZnO(0001) surface. The charges on the nearest O and Zn atoms are also shown. In this figure, light gray, red, dark gray, and yellow spheres represent Rh, O, Zn, and Ir atoms, respectively.

In this study, we considered two methods of introducing the single-atom component to the support surface: substitutional and adsorbed. Starting with a perfect surface, we first substituted a Rh or Ir atom in place of a Zn atom on the 2×2 surface. Beyond stabilizing the single atom, the new coordination environment arising from substitutional incorporation of a metal atom would also modify its charge state and the position of its *d* orbitals, possibly altering its reactivity. Upon geometry relaxation, both the Rh and the Ir atoms were seen to undergo a noticeable elevation with respect to the original position of Zn, as illustrated in Figure 1.

This elevation was even more pronounced in the case of Rh. A Bader charge decomposition analysis, also shown in the Figure 1, reveals that upon adsorption, Rh and Ir attained partial charges of +0.43 |e| and +0.34 |e|, respectively.

**Figure 2:**
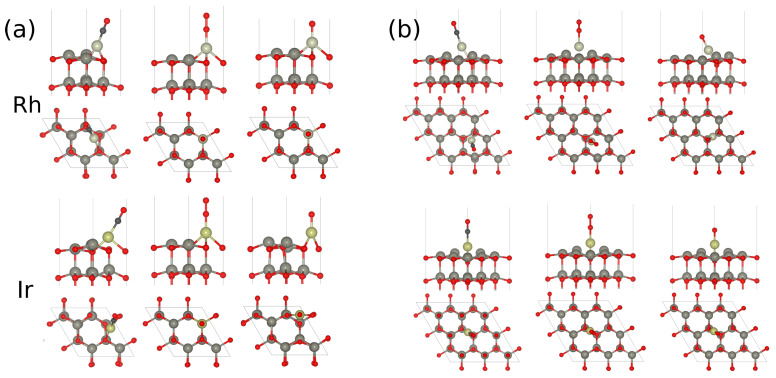
Optimized CO, O_2_, and O adsorption geometries on the 2×2 (**a**) and 3×3 (**b**) ZnO(0001) surfaces. In this figure, light gray, red, black, dark gray, and yellow spheres represent Rh, O, C, Zn, and Ir atoms, respectively.

Next, we investigated the adsorption characteristics of the relevant species involved in the CO oxidation reaction, namely CO, O_2_, and O. In actual experimental settings, there may be a wide variety of SAC coverages. For instance, Xue et al. [75] investigated Pt loadings on CdS nanosheets ranging from 0.3% to 3% weight. HAADF-STEM images of their loading clearly show several instances of metal atoms that are no further than a few lattice sites. Since lateral interactions between adsorbates can significantly influence adsorption behavior [76], we first assessed the importance of coverage, in preparation for the rest of our results. To this end, we considered adsorption in both a 2×2 (1/4 coverage) and a 3×3 (1/9 coverage) periodic surface cell. The optimized CO, O_2_, and O adsorption geometries, energies, and partial charges are reported in Figure 2 and Table 1 for these coverages.

For the most part, Rh and Ir yielded similar results for all the properties displayed in Table 1, with molecular adsorption energies being slightly higher on Ir with respect to Rh. A notable exception is that Rh significantly underbound all three adsorbates in the 2×2 cell with respect to Rh in the 3×3 cell (see the paragraph below). Consequently, at 1/4 coverage, Ir resulted in a larger adsorption energy than Rh by 1.1 eV (40.5%), 0.5 eV (34.0%), and 1.9 eV (40.0%) for CO, O_2_, and O, respectively. For the 1/9 coverage, the difference was much less pronounced, with Ir still binding the adsorbated more strongly, but by only 15.7%, 3.0%, and 10.3%. The stretch mode frequencies were largely independent of coverage for CO. However, for both O_2_ and O, the 3×3 coverage gave significantly lower frequencies, in spite of the almost identical bond lengths. Frequencies in the presence of Ir were higher.

The PDOS plots and charge density difference contours displayed in Figures S1–S3 of the Supplementary Materials (SMs) shed light on the adsorption results. For CO adsorption (Figure S1), the donation–back donation mechanism often attributed to metal–CO binding [62] can also be clearly seen from the CDD contours. While the general picture was the same in all cases, a particularly clear example can be seen in Figure S1d, where the CO adsorbed on Ir is surrounded by a red region of charge deficiency which has the form of the CO HOMO orbital, while the blue rings around both C and the O atoms correspond to the CO LUMO orbital. Simultaneously, charge shifts also occur internally within Ir between different *d* orbitals. Overall, this resulted in a charge accumulation between Ir and C forming a bond while the C–O bond weakened due to charge loss. This behavior did not show appreciable variation between the two coverages or the two metals. The PDOS profiles and Bader charges, however, showed significant differences that could explain the trends we have outlined above. For CO adsorption in the 2×2 model, the Fermi level coincided with a spin-split orbital of mostly Rh *d*-character. The lifting of this spin degeneracy caused this state to be partially occupied and the deeper CO levels to shift up by about 2 eV towards the Fermi level with respect to the 3×3 slab. This was consistent with the much larger Rh Bader charge and the much smaller CO adsorption energy for the 1/4 coverage given in Table 1. The total charge transferred to the CO molecule was very close for the two coverages and the two metals.

O_2_ can adsorb on metal substrates both in the side-on and end-on configurations [77,78]. Here, we placed the O_2_ molecule in a perpendicular orientation at the beginning of the geometry optimization. All optimization processes terminated with O_2_ in the end-on geometry. The O_2_ molecule was activated on transition metal centers and substrates via transfer of electrons to one (the superoxo state) or two (the peroxo state) of its $\pi^{*}$ orbitals from the metal *d* orbitals. This was accompanied by a lengthening of the bond length and decrease in the stretch mode frequency [79,80]. The results in Table 1 indicate that for both coverages and both metals, there was indeed a charge transfer of about 0.4–0.5 |e| from the metal to O_2_. Similarly to the case of CO, the metals in the 2×2 cells donated about 0.3 |e| more than those in the 3×3 cells. However, the adsorbate charge was almost the same for both metals and both coverages. This means that the difference in the donated charge was transferred to the rest of the atoms on the surface rather than the adsorbate. In the gas and the superoxo phases, the O-O bond length was 1.21 Å and 1.33 Å, respectively. The calculated bond length in the O_2_ adsorbate here was slightly larger than the gas phase, between 1.27 Å and 1.29 Å. The metals in the 1/9 coverage yielded slightly larger bond lengths and significantly lower frequencies. The CDD contours and PDOS profiles displayed in Figure S2 for both metals in the 1/4 coverage were similar. The hybrid orbital formed through the mixing of the O_2_ $\pi^{*}$ and the metal *d* orbital had a slight occupation, which accounted for the charge gain. In the 3×3 systems, this orbital was slightly split into above and below the Fermi level since it had a higher occupation. This may explain the higher adsorption energies. This state and the lower states were significantly spin-polarized for Ir at this coverage, with a total magnetization of 3.3 $\mu_{B}$. All CDD clearly demonstrate the depletion of charge from the metal *d* state to the O_2_ antibonding state.

Finally, the strongest adsorption among the three adsorbates studied here belonged to atomic O. The chemical bonds between all 3d, 4d, and 5d transition metals and atomic oxygen were studied at the CCSD(T) level by Moltved and Kepp [81]. In this study, both Rh and Ir, being late transition metals, were shown to have a mid-range affinity towards O, with metal–O bond dissociation enthalpies of 4.25 eV and 4.69 eV for RhO and IrO, respectively. These results are consistent with our findings. In the same study, the charge transfer from the metal to the O atom was reported as around 0.5 |e| for both metals. In our results, the O atom had a slightly higher charge for both metals and both coverages, which was about 0.6 |e|. The charge on the metal, on the other hand, was once again coverage-dependent, with the 1/4 coverage resulting in a higher positive charge. The CDD contours in Figure S3 clearly show the excess charge on the O atom. All PDOS plots indicate highly spin-polarized states, as expected [81].

Our findings regarding coverage so far highlight two important points. The first is that coverage definitely affects some adsorption properties, and is therefore important to consider in future calculations. And secondly, the marked difference in the stretching frequency may be used as a viable tool to experimentally distinguish the two coverages. In the rest of our work, we used the 2×2 cells for our calculations.

### 3.2. Rh and Ir Adsorbed on Impurity-Doped ZnO(0001)

**Figure 3:**
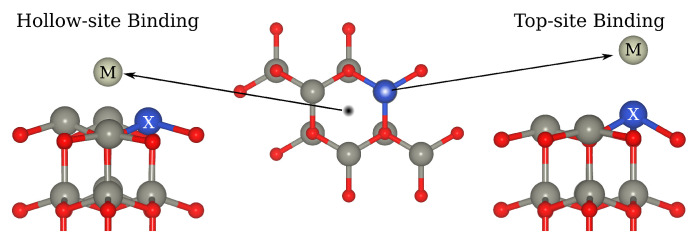
Top and side-view illustration of hollow and top binding geometries of single metal Ir and Rh on the impurity-enriched ZnO(0001) surface. X = Rh, Ir, Al, Cr, Cu, Pd; M = Rh, Ir. In this figure, dark gray, red, navy blue, and yellow spheres represent Zn, O, promoter metals, and Rh/Ir atoms, respectively.

In this section, we present our results on single-atom Rh and Ir on the Zn(0001) surface already decorated by Rh, Ir, Al, Cr, Cu, and Pd impurities. Figure 3 displays the two geometries tested during the investigation of the most favorable adsorption site of the Rh and Ir single atom. The substitutional doping metal was placed at the location of a Zn that had been removed from the surface (labeled X in the Figure), while the Rh or Ir atom (labeled M in the figure) was placed either directly on top of X or on an adjacent hollow site. The Rh and Ir adsorption energies for each doping metal and each location are listed in Table 2.

For both metals, the hollow sites yielded significantly larger adsorption energies. Ir bound more strongly to all impurities than Rh in both geometries. The Rh-Rh and Ir-Ir bindings are included in our results to assess the strength of the bimetallic bond, which is important for understanding the likelihood of segregation by means of forming small clusters. Both the Rh_2_ and the Ir_2_ had substantially large binding energies (among the largest presented in the Table 2), indicating that clustering was indeed an important issue to consider here.

An immediately apparent result was that in contrast to the unpromoted ZnO surface, both Rh and Ir acquired a partial negative charge. This was likely to affect the charge transferred to the molecular species, thereby changing their activation towards the reaction.

Comparing different impurities (other than Rh_2_ and Ir_2_), both Rh and Ir bound the most strongly on Al and Cr. Consistent with the binding strength, interaction with Al and Cr also led to the highest negative charge accumulation on Ir and Rh. This illustrates the synergistic interaction between the metal oxide surface and the impurity atom, which collectively influenced the partial charges on Rh and Ir. The surface governed the charge on the impurity, which, in turn, modulated the charge on the metal atom. In the comprehensive experimental and theoretical study by Hulva et al. [62] regarding CO adsorption on iron oxide-supported transition metals with different *d* orbital populations, it was demonstrated that as the population of *d* orbitals increased, the backdonation from the metal *d* orbital to the CO 2$\pi^{*}$ orbital increased as well. This was expected to further weaken the CO bond. Therefore, since surface doping with Al and Cr resulted in the largest charge transfer to Ir and Rh, we isolated them and studied the adsorption characteristics of CO, O_2_, and O on the single metals paired with these two impurities.

Before studying the adsorbed molecules, we considered the metal–metal interaction by itself. Interestingly, from the CDD contours presented in Figure S4 for the Rh-Al, Rh-Cr, Ir-Al, and Ir-Cr pairs, one observes an accumulation of charge in the region between the two metals. The PDOS profiles presented in the same figure reveal that the coupling of the Al orbitals with Rh and Ir was rather weak, forming an orbital at the Fermi level with contributions mostly from the *d* electrons of the active metal with minor contrbutions from Al, Zn, and O. Interaction with Cr caused a strong spin polarization of Rh and Ir.

**Figure 4:**
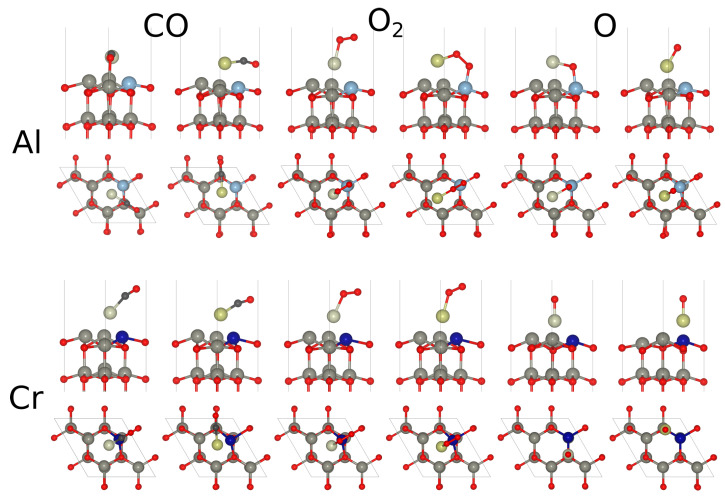
Optimized adsorption geometries of CO, O_2_, and O on Rh and Ir placed on Al (top panels)- and Cr (bottom panels)-decorated ZnO(0001) surfaces. In this figure, blue, dark blue, dark gray, black, red, light gray, and yellow spheres represent Al, Cr, Zn, C, O, Rh, and Ir atoms, respectively.

The optimized adsorption geometries are shown in Figure 4 and the adsorption characteristics are reported in Table 3. In all the systems considered below, Rh and Ir were placed at the hollow sites. The geometry optimization for all adsorption geometries were initiated by placing the adsorbate in an orientation perpendicular to the surface on top Rh or Ir. During the optimization process, however, all adsorbates oriented themselves to varying degrees, towards the surface with the exception of O on the Rh/Cr and Ir/Cr systems, which remained in an on-top position.

From the data listed in Table 3, it is immediately clear that since Al binds both Rh and Ir very strongly (see Table 2) it weakens the C–O bond. The C–O bond distance was larger than any other case reported in this work, while the vibration frequency was significantly lower. The C–O bond length and vibrational frequency were instead not much affected by the presence of Cr. For O_2_, the promoting effect of the impurity was not at all evident with low adsorption energies, bond lengths close to gas phase, and large negative charges on both the noble metal and O_2_. One significant exception is the O_2_ adsorption geometry on the Al-Ir pair, where the molecule formed a perfect bridge between the two metals. In this case, the bond length was as large as 1.50 Å, which was consistent with the bond distance of the peroxo state, reported by Holland [79]. In this activated state, O_2_ scission would occur easily. Finally, Al also yielded a higher binding energy than Cr in the case of Rh for the single O atom. However, as an exception to the trend, Cr worked somewhat better when paired with Ir. From Table 3, one notices that the stretch mode frequencies of the adsorbates show a depencence on the impurity present on the ZnO surface. In correlation with the bond length variations, Cr yielded higher frequencies with differences approaching 200 ${\mathrm{cm}}^{-1}$. This also applies to O, while the differences (excluding the instance of dissociative adsorption) for O_2_ were less pronounced.

**Figure 5:**
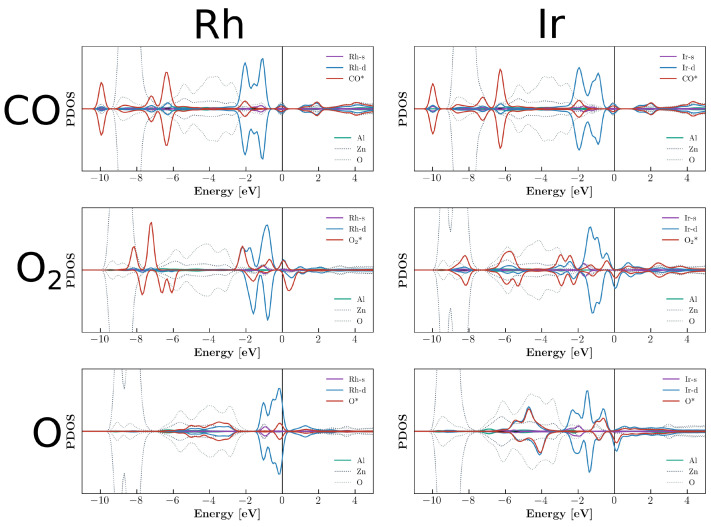
Partial density of states of CO, O_2_, and O adsorption on Rh and Ir on the Al-decorated ZnO(0001) surface.

**Figure 6:**
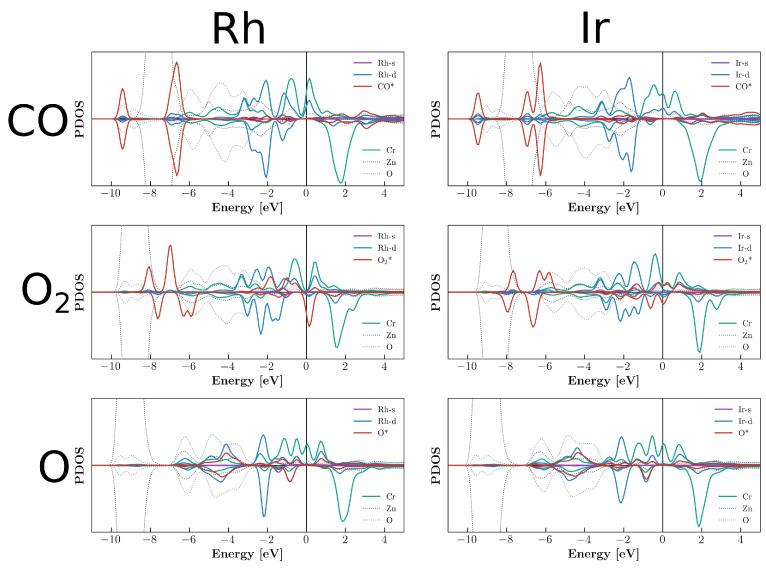
Partial density of states of CO, O_2_, and O adsorption on Rh and Ir on the Cr-decorated ZnO(0001) surface.

PDOS plots for adsorption with impurities are shown in Figure 5 and Figure 6. These profiles clearly display the difference between the mode of operation of Al and Cr as promoters. In the case of Al, the contribution was mostly indirect by means of changing the charge state of Rh. The Al contributions were very small for the electronic states near the Fermi level. In the case of Cr, due to the large number of electrons and the highly magnetic nature, the interaction was dominated by the Cr states. The systems were highly spin-polarized with a large *d* electron contribution from Cr near the Fermi level. The related CDD plots are given in Figure S5.

We also considered the adsorption of the CO oxidation product, CO_2_, on the impurity-enriched SACs. In Figure S6 of the SM, we display the CDD contours and PDOS profiles of CO_2_ adsorption on Rh and Ir on Al-decorated Zn(0001). In both cases, the geometry is one where an oxygen atom of the adsorbate binds a surface Zn atom and the C points towards Rh or Ir. The CO_2_ adsorption energies are −1.54 eV and −1.60 eV for Rh and Ir, respectively. The M-C distances (M = Rh, Ir) in both cases are around 1.9 Å. The Rh and Ir partial charges are −0.49 |e| and −0.61 |e|, respectively, while the charges on CO_2_ are −0.65 |e| for both metals. As expected, the negative charge state on the CO_2_ molecule caused the molecule to bend. The CDD contours indicate a clear bond formation via charge accumulation in the Zn-O and M-C regions. The PDOS plots are very similar where near the Fermi energy, metal *s*, metal *d*, CO_2_, and Al *s* states overlap.

## 4. Conclusions

In this study, we investigated the electronic structure of ZnO-supported single Rh and Ir atoms and their activity towards CO oxidation using density functional theory-based calculations and analysis tools. As indicators, we used adsorption energies, Bader partial charges, vibrational frequencies, and partial density of states analysis for the adsorption of CO, O_2_, and O species. Properties of adsorption on cationic substitutional Rh and Ir atoms somewhat depended on coverage. The positive Bader charges on Rh and Ir atoms in a 2×2 surface cell were larger, and as a result, all adsorbates interacted less strongly. For both coverages, however, the CO bond was lengthened compared to the value in the gas-phase and the O_2_ molecule was activated.

As a further step, we considered Rh and Ir single atoms on ZnO(0001) surfaces promoted by other metal atoms. Of the six screened promoters (Rh, Ir, Al, Cr, Cu, and Pd), Al and Cr yielded the largest Ir and Rh binding energies as well as Bader charges. In contrast to the Zn-substituted Rh and Ir, the Bader charges on both metals on promoted surfaces were negative. Upon CO adsorption, this extra *d*-orbital population on the metals resulted in the largest charge transfer to the molecular antibonding $\pi^{*}$ orbital, weakening the C–O bond. Simultaneously, Al-promoted Ir was also seen to donate electronic charge to O_2_ and thereby activate it. Both cationic substitutional and promoted Rh and Ir on ZnO(0001) have the potential to be active in CO oxidation. In further works, it would be interesting to study in detail how the charge state of Rh and Ir species influences their catalytic activity towards reactions of current interest.

## Figures and Tables

**Table 1:** Adsorption energies ($E_{b}$), Bader charges (*q*), vibrational frequencies (*f*), and bond distances (*d*) of CO, O_2_, and O adsorption on 2×2 and 3×3 Rh- and Ir-decorated ZnO(0001). The subscript *X* represents either Rh or Ir, while *A* and *B* correspond to the individual atoms of the adsorbate, respectively. A* designates the atom directly in contact with the surface (e.g., in the case of CO, A* is C.) The stretch mode frequencies reported for O correspond to the O-X bond.

	CO				O_2_				O			
	2×2		3×3		2×2		3×3		2×2		3×3	
	Rh	Ir	Rh	Ir	Rh	Ir	Rh	Ir	Rh	Ir	Rh	Ir
$E_{b}$ [eV]	−2.74	−3.85	−3.43	−3.97	−1.47	−1.97	−2.01	−2.07	−4.77	−6.68	−5.80	−6.40
$q_{X}$ [|e|]	0.44	0.41	0.04	0.07	0.64	0.71	0.37	0.30	0.93	0.78	0.49	0.42
$q_{A^{*}}$ [|e|]	0.88	0.87	0.88	0.84	−0.29	−0.34	−0.24	−0.33	−0.61	−0.61	−0.62	−0.64
$q_{B}$ [|e|]	−1.10	−1.10	−1.11	−1.11	−0.06	−0.07	−0.18	−0.13	–	–	–	–
$d_{A-B}$ [Å]	1.17	1.17	1.17	1.18	1.27	1.28	1.29	1.29	–	–	–	–
f [${\mathrm{cm}}^{-1}$]	1968.63	1985.14	1959.92	1963.94	1323.51	1331.73	1208.50	1234.77	851.63	908.51	793.41	870.07

**Table 2:** Binding energies and metal/impurity partial Bader charges for substitutional impurities in the two geometries illustrated in Figure 3. In the upper half of the table, the metal under consideration is Rh, whereas in the lower half, it is Ir. The subscript *X* in $q_{X}$ represents the impurities.

	Top					Hollow				
M = Rh	Rh	Al	Cr	Cu	Pd	Rh	Al	Cr	Cu	Pd
$E_{b}$ [eV]	−3.94	−3.58	−3.79	−1.81	−2.38	−4.28	−4.99	−4.74	−2.74	−3.34
$q_{X}$ [|e|]	0.44	2.24	1.24	0.54	0.22	0.69	2.25	1.31	0.74	0.51
$q_{M}$ [|e|]	−0.11	−0.84	−0.45	−0.12	0.03	−0.27	−0.82	−0.52	−0.23	−0.21
M = Ir	Ir	Al	Cr	Cu	Pd	Ir	Al	Cr	Cu	Pd
$E_{b}$ [eV]	−4.68	−4.10	−4.67	−2.43	−3.15	−5.80	−5.88	−5.78	−3.47	−4.16
$q_{X}$ [|e|]	0.33	2.29	1.24	0.61	0.29	0.70	2.29	1.31	0.78	0.54
$q_{M}$ [|e|]	−0.09	−1.02	−0.61	−0.27	−0.07	−0.39	−0.99	−0.65	−0.39	−0.32

**Table 3:** Adsorption energies ($E_{b}$), Bader charges (*q*), vibrational frequencies (*f*), and bond distances (*d*) of CO, O_2_, and O on Rh and Ir placed on ZnO(0001) with substitutional Al and Cr. The subscript X stands for Rh or Ir, and Y for Al or Cr, while A and B correspond to the individual atoms of the adsorbate, respectively. The subscript (∗) indicates the atom of the adsorbate directly attached to the surface. The stretch mode frequencies reported for O correspond to the O-Y bond.

	CO				O_2_				O			
	Rh–Al	Rh–Cr	Ir–Al	Ir–Cr	Rh–Al	Rh–Cr	Ir–Al	Ir–Cr	Rh–Al	Rh–Cr	Ir–Al	Ir–Cr
$E_{b}$ [eV]	−2.16	−0.71	−2.50	−1.21	−0.28	−0.11	−1.91	−0.24	−5.71	−4.13	−4.24	−4.80
$q_{X}$ [|e|]	−0.49	−0.35	−0.60	−0.37	−0.49	−0.20	0.02	−0.29	0.07	0.06	−0.28	0.05
$q_{Y}$ [|e|]	2.30	1.32	2.34	1.35	2.26	1.31	2.42	1.30	2.43	1.26	2.31	1.27
$q_{A^{*}}$ [|e|]	0.77	0.93	0.75	0.89	−0.19	−0.24	−0.33	−0.27	−1.12	−0.56	−0.57	−0.63
$q_{B}$ [|e|]	−1.18	−1.08	−1.17	−1.12	−0.11	−0.12	−0.78	−0.12	–	–	–	–
$d_{A-B}$ [Å]	1.20	1.16	1.21	1.17	1.28	1.29	1.50	1.29	–	–	–	–
*f* [${\mathrm{cm}}^{-1}$]	1728.48	1990.71	1730.06	1940.57	1246.31	1247.60	695.95	1211.55	660.75	880.38	894.30	937.90

## Data Availability

The data that support the findings of this study are available from the corresponding author upon reasonable request.

## Supplementary Materials

The following supporting information can be downloaded at https://www.mdpi.com/article/10.3390/molecules29215082/s1, Figure S1: Charge density difference (CDD) contours and partial density of states (PDOS) of CO adsorbed on 2 × 2 and 3 × 3 ZnO(0001) with substitutional Rh (a),(c), and Ir(b),(d). In the CDD plots red and blue regions correspond to electron deficiency and excess, respectively. Figure S2: Charge density difference (CDD) contours and partial density of states (PDOS) of O_2_ adsorbed on 2 × 2 and 3 × 3 ZnO(0001) with substitutional Rh (a),(c), and Ir(b),(d). In the CDD plots red and blue regions correspond to electron deficiency and excess, respectively. Figure S3: Charge density difference (CDD) contours and partial density of states (PDOS) of O adsorbed on 2 × 2 and 3 × 3 ZnO(0001) with substitutional Rh (a),(c), and Ir(b),(d). In the CDD plots red and blue regions correspond to electron deficiency and excess, respectively. Figure S4: Charge density difference (CDD) contours and partial density of states (PDOS) of Rh and Ir adsorbed on substitutionally doped Al and Cr on the Zn(0001) surface. In the CDD plots red and blue regions correspond to electron deficiency and excess, respectively. Figure S5: Charge density difference (CDD) plots for CO, O_2_, and O adsorption on Rh and Ir on the Al- and Cr-decorated ZnO(0001) surface along with PDOS profiles. In the CDD plots red and blue regions correspond to electron deficiency and excess, respectively. Figure S6: Charge density difference (CDD) plots for O_2_ adsorption on Rh and Ir on the Al-decorated ZnO(0001) surface along with PDOS profiles. In the CDD plots, the blue regions indicate charge excess and the red regions charge depletion.

## Abbreviations

The following abbreviations are used in this manuscript:

SACs	Single-atom catalysts
DFT	Density Functional Theory
PBE	Perdew–Burke–Ernzerhof exchange correlation potential
PAW	projector-augmented-wave scheme
PDOS	Partial Density of States
CDD	Charge density difference
SM	Supplementary Material
