# Supplementary material for: Electronic Structure of Rh and Ir Single Atom Catalysts Supported on Defective and Doped ZnO: Assessment of Their Activity Towards CO Oxidation
Source: Molecules. 2024 Oct 28;29(21):5082. doi: 10.3390/molecules29215082 (PMC11547260; doi:10.3390/molecules29215082)
Supplement: Supplementary file 1 [file molecules-29-05082-s001.zip › molecules-3235096-supplementary.pdf]

Supporting information for: Electronic Structure of Rh and Ir Single Atom Catalysts Supported on Defective and Doped ZnO: Assessment of Their Activity Towards CO Oxidation.

Arda Erbasan,† Hande Ustunel,*,† and Daniele Toffoli*,‡,¶

†*Department of Physics, Middle East Technical University, Dumlupinar Blv 1, 06800, Ankara, Turkey*

‡*Dipartimento di Scienze Chimiche e Farmaceutiche, Università degli Studi di Trieste, Via L. Giorgieri 1, I-34127, Trieste, Italy*

¶*IOM-CNR, Istituto Officina dei Materiali-CNR, S.S.14, km 163.5, 34149 Trieste, Italy*

E-mail: ustunel@metu.edu.tr; toffoli@units.it

(a) 2×2 ZnO-Rh-CO

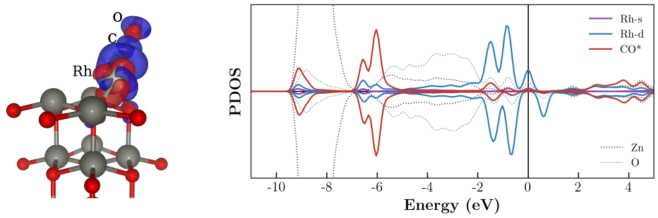

(b) 2×2 ZnO-Ir-CO

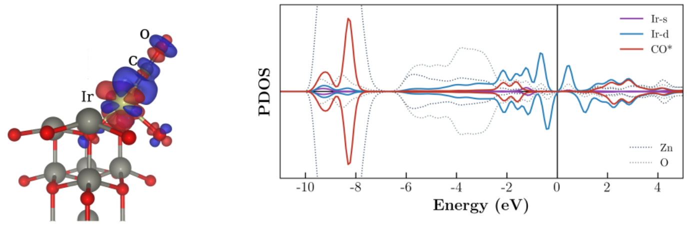

(c) 3×3 ZnO-Rh-CO

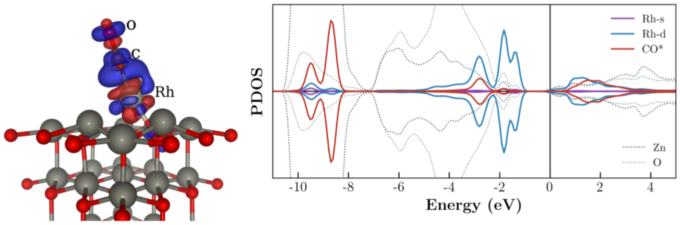

(d) 3×3 ZnO-Ir-CO

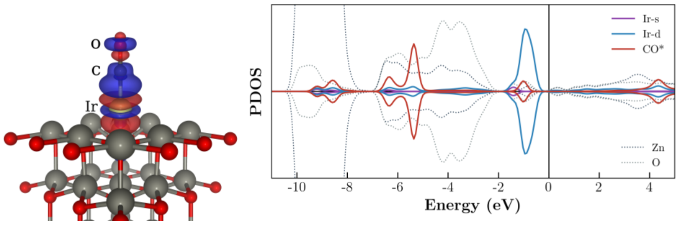

Figure S1: Charge density difference (CDD) contours and partial density of states (PDOS) of CO adsorbed on 2×2 and 3×3 ZnO(0001) with substitutional Rh (a),(c), and Ir(b),(d). In the CDD plots red and blue regions correspond to electron deficiency and excess, respectively.

(a) 2×2 ZnO-Rh-O_2

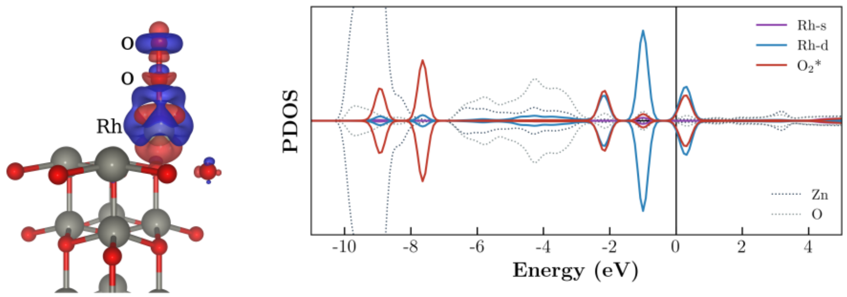

(b) 2×2 ZnO-Ir-O_2

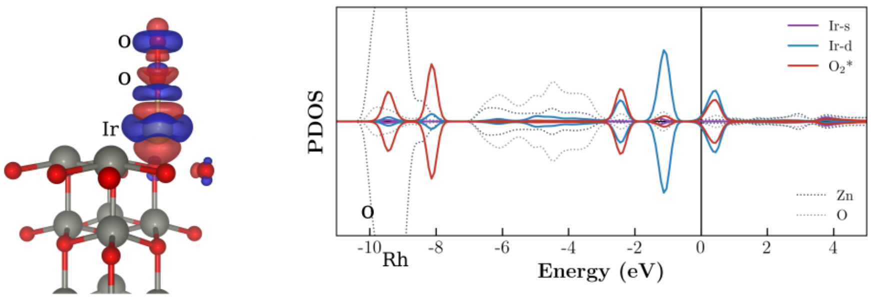

(c) 3×3 ZnO-Rh-O_2

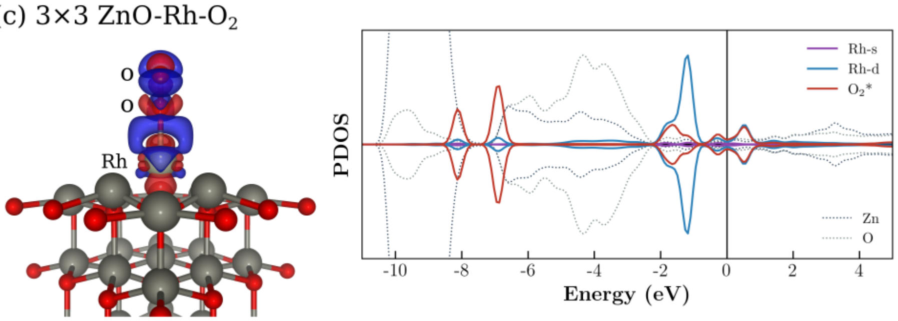

(d) 3×3 ZnO-Ir-O_2

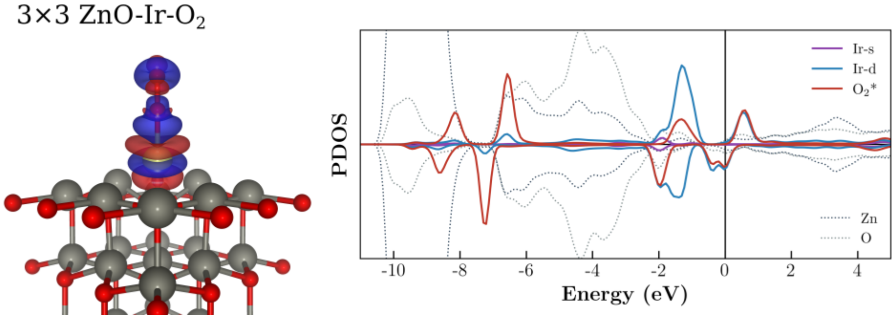

Figure S2: Charge density difference (CDD) contours and partial density of states (PDOS) of O_2 adsorbed on 2×2 and 3×3 ZnO(0001) with substitutional Rh (a),(c), and Ir(b),(d). In the CDD plots red and blue regions correspond to electron deficiency and excess, respectively.

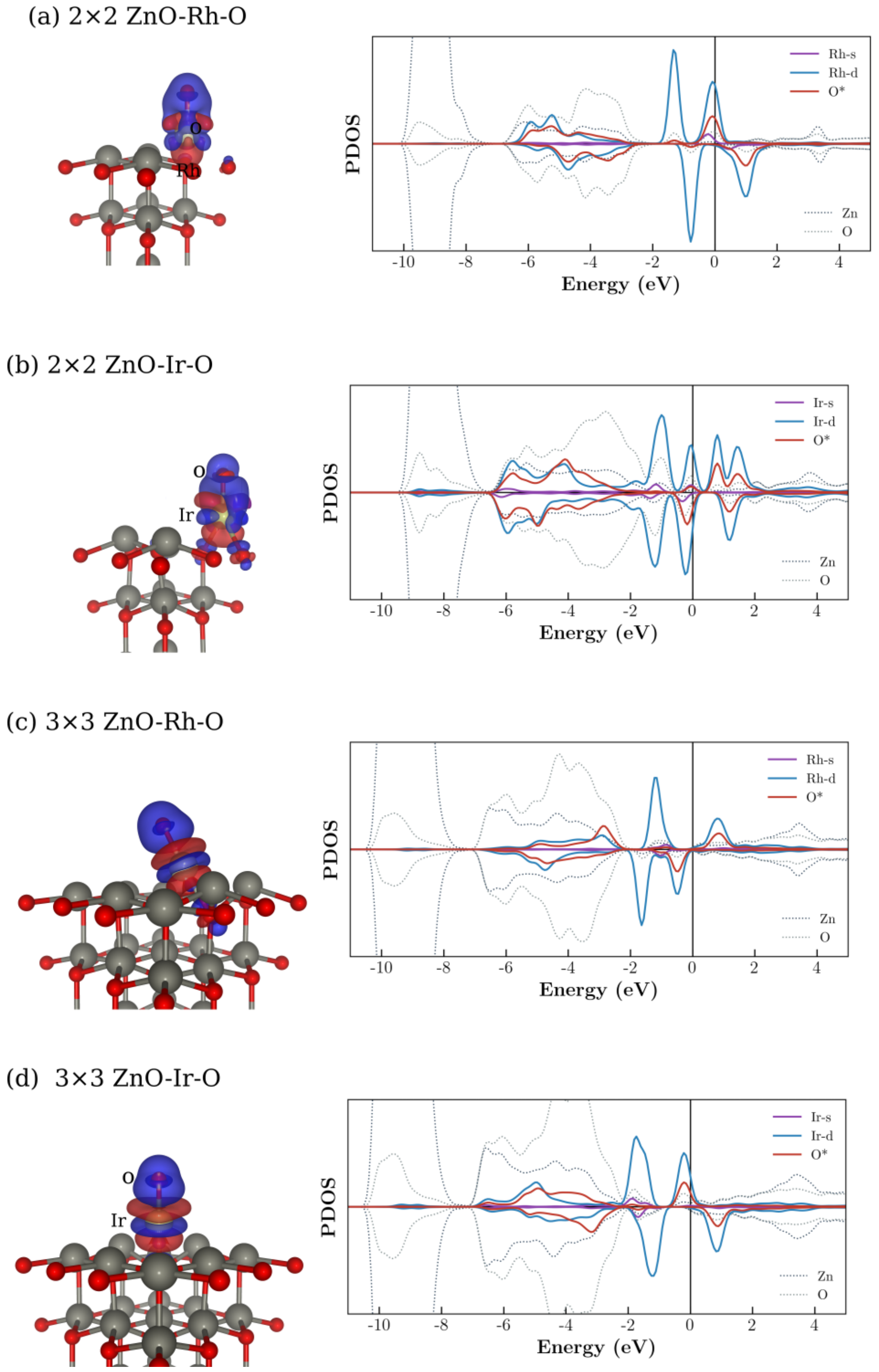

Figure S3: Charge density difference (CDD) contours and partial density of states (PDOS) of O adsorbed on 2×2 and 3×3 ZnO(0001) with substitutional Rh (a),(c), and Ir(b),(d). In the CDD plots red and blue regions correspond to electron deficiency and excess, respectively.

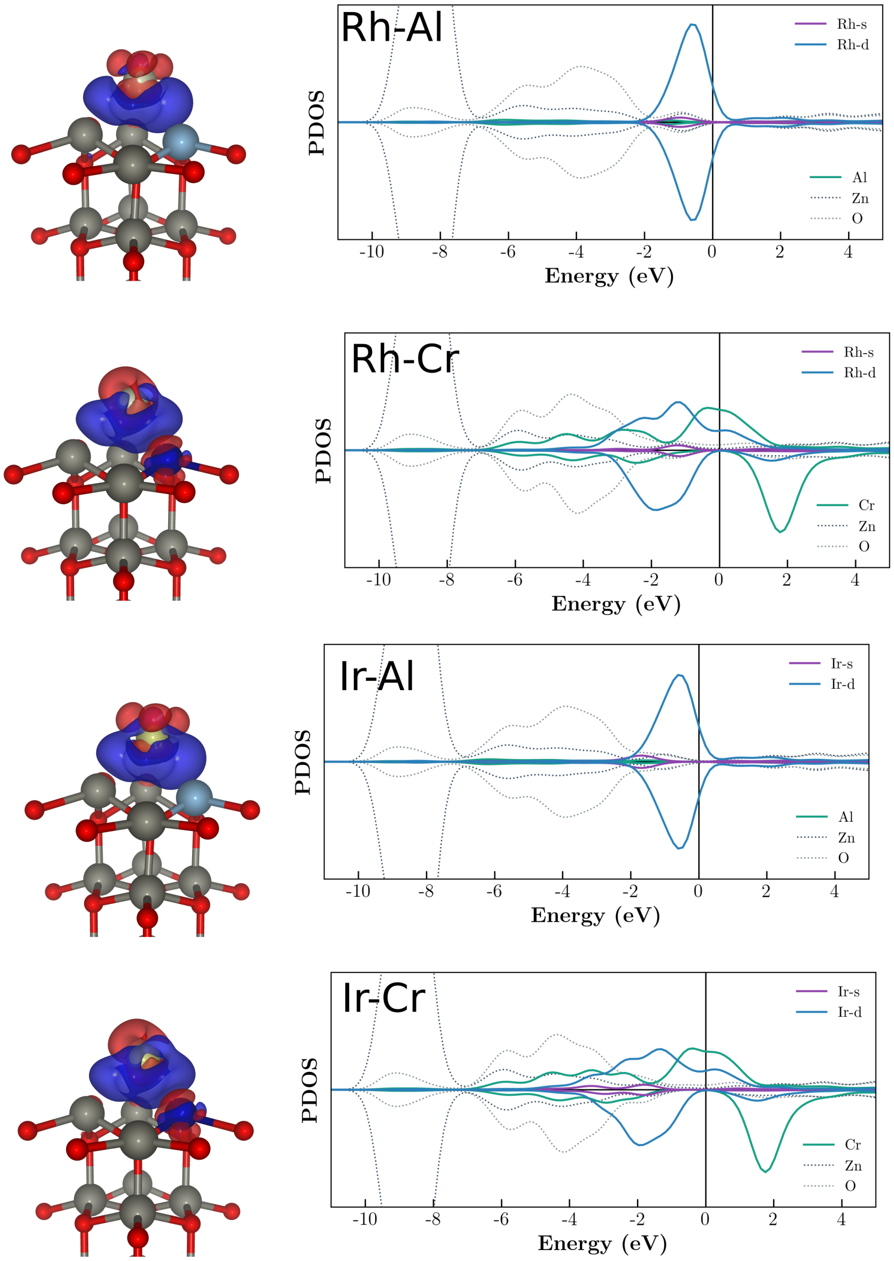

Figure S4: Charge density difference (CDD) contours and partial density of states (PDOS) of Rh and Ir adsorbed on substitutionally doped Al and Cr on the Zn(0001) surface. In the CDD plots red and blue regions correspond to electron deficiency and excess, respectively.

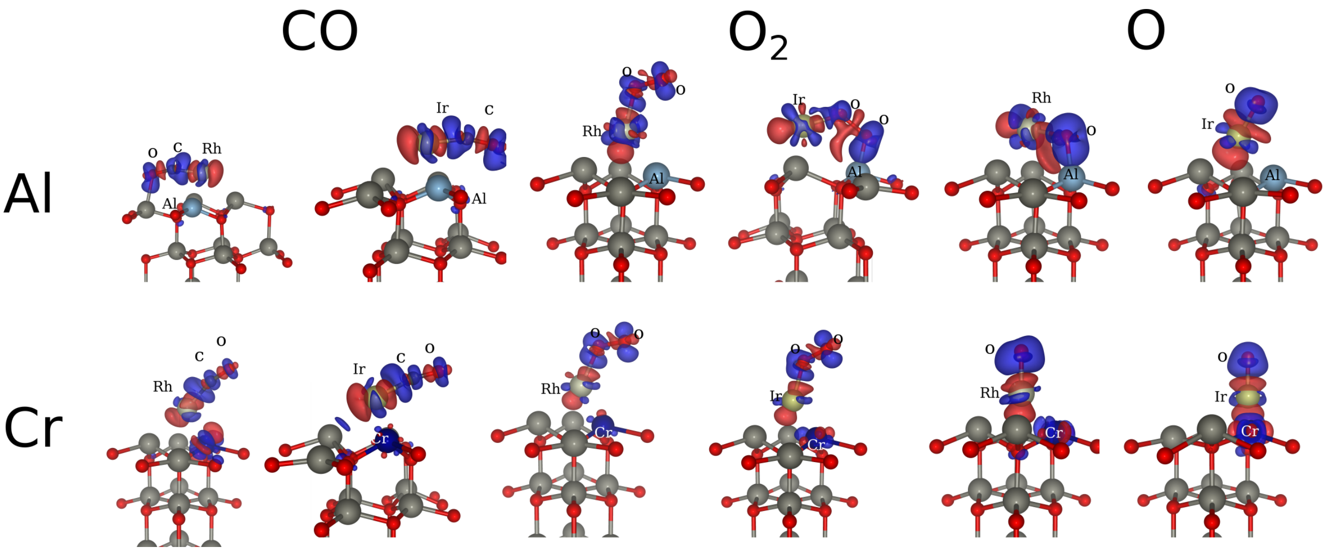

Figure S5: Charge density difference (CDD) plots for CO, O_2, and O adsorption on Rh and Ir on the Al- and Cr-decorated ZnO(0001) surface along with PDOS profiles. In the CDD plots red and blue regions correspond to electron deficiency and excess, respectively.

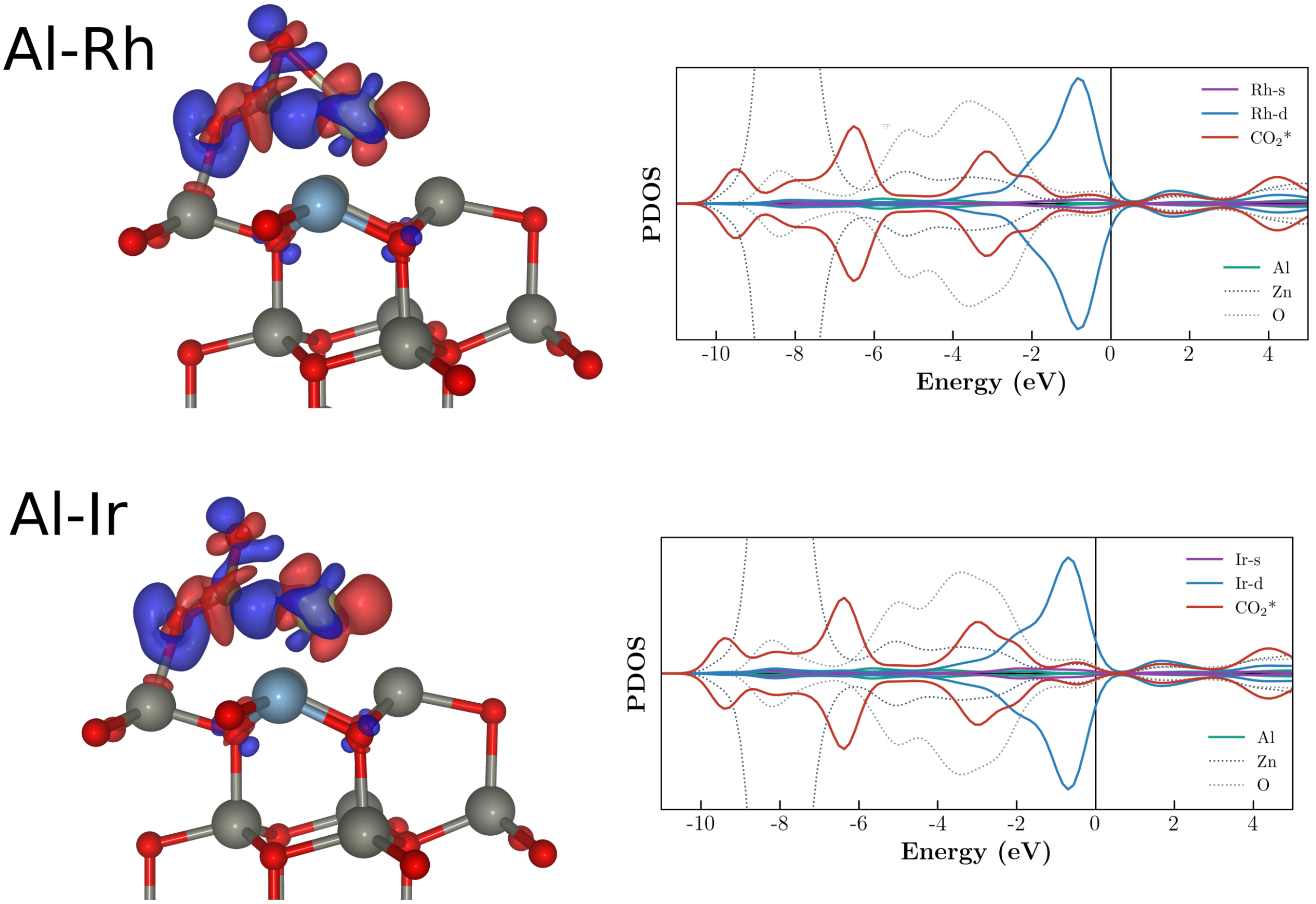

Figure S6: Charge density difference (CDD) plots for CO_2 adsorption on Rh and Ir on the Al-decorated ZnO(0001) surface along with PDOS profiles. In the CDD plots, the blue regions indicate charge excess and the red regions charge depletion.
